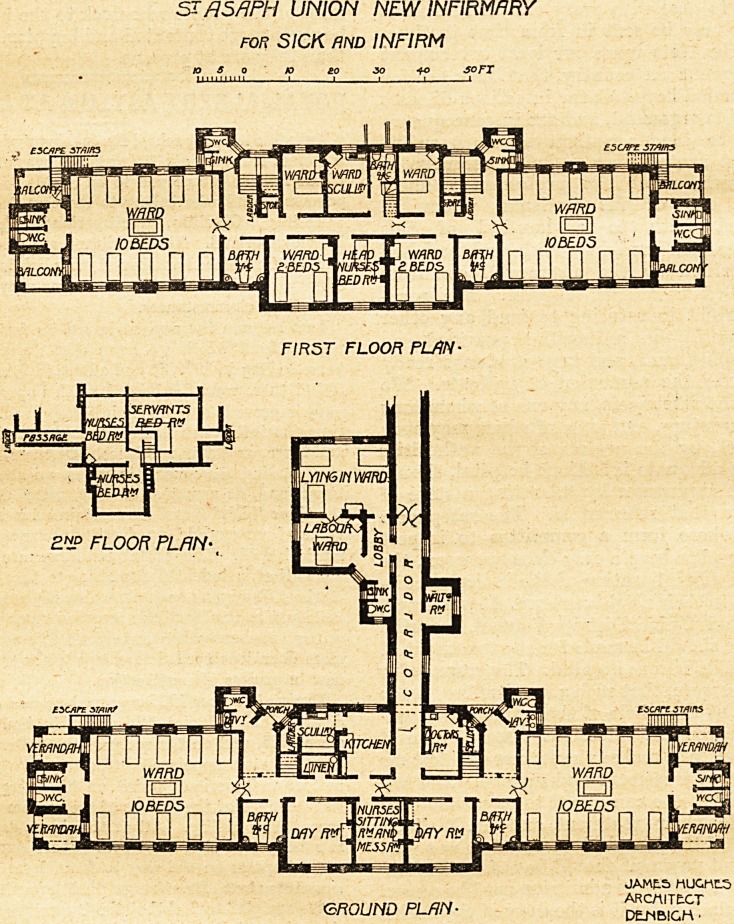# St. Asaph Union Workhouse Infirmary

**Published:** 1907-01-26

**Authors:** 


### St. ASAPH UNION WORKHOUSE INFIRMARY.

This infirmary was formally opened early in the year by the Chairman of the Board of Guardians, Mr. T. Howes Roberts. It consists of two blocks, which are connected with each other by a corridor and with the main building by a covered way. The larger block is of linear design, formed by a centre and two wings. On the ground floor the centre to the front contains a nurses' sitting-room and mess-room, and having a day-room on each side. Behind these is a corridor which cuts the centre in two, and gives access to the main wards and to the various rooms forming the centre part of the block. This corridor is nearly sixty feet long, and, judging from the plans sent to us, we should doubt whether it is sufficiently lighted. At the opposite side are the kitchen, scullery, staircases, lobbies, medical officers' room, lavatories and closets. Between the kitchen and the doctor's room is the corridor which connects the block with the workhouse.

The bath-rooms are placed between the day-rooms and the large wards. These large wards, of course, constitute the greater part of the wings. They contain ten beds each, are about 33 feet long by 25 feet wide, and therefore each patient will have something over 800 square feet of floor space. Excepting those beds nearest the centre, each bed has a window on both sides. At the ends of the wards are placed the closets and sinks. They project from the ward, and are properly arranged and satisfactorily cut off from the main by cross-ventilated passages. They are flanked by small verandahs, and from one of these springs the fire-escape staircase.

The disposition of the first floor is necessarily very similar to the ground floor. The space over the day-rooms is made into two-bedded rooms, the mess-room into head nurse's bedroom, and there are two single-bedded wards, ward scullery and bath-room placed over the kitchen department.

Taking it as a whole, and bearing in mind that it is a workhouse infirmary, it may be said that the building is well enough designed and arranged, and it contains some good points, as, for instance, the verandahs and the balconies. On the other hand, the two-bedded wards and the single-bedded wards are insufficiently cross-ventilated, although we notice that the former have windows in the wall between them and the corridor. Still it is only ventilation into a corridor. Unless the doors be left open the single-bedded rooms have no cross-ventilation at all, but they may have fanlights. It must be admitted that the fault is a very common one, and, in small hospitals at any rate, it is not always easy to steer clear of it.

The maternity block is placed between the infirmary and the main building—a very good position for it. The block contains a three-bedded room, a single-bedded room, a waiting-room and the usual sanitary arrangements. The block is compact and well arranged.

The infirmary is built of limestone, with a lining of brick, and the wards, passages and rooms have a dado of glazed bricks. Internal angles have been rounded off. The wards are warmed by Musgrave stoves, and fresh air is admitted by Tobin's tubes. There are foul-air shafts running above the roof and connected with air-pump extractors. The drainage is into the main sewer of the city.

The cost was £8,000, or £160 per bed, a sum which must be considered moderate when it is known that it includes electric light installation for the whole workhouse, furniture for the infirmary, boundary walls, drainage and architect's fees.

The architect was Mr. James Hughes, of Denbigh, and the contractors were Messrs. Evans and Son, of Colwyn.

## Figures and Tables

**Figure f1:**